# Trends in Breast Cancer Mortality in Sweden before and after Implementation of Mammography Screening

**DOI:** 10.1371/journal.pone.0022422

**Published:** 2011-09-26

**Authors:** Jari Haukka, Graham Byrnes, Mathieu Boniol, Philippe Autier

**Affiliations:** 1 Faculty of Medicine, Department of Public Health, University of Helsinki, Helsinki, Finland; 2 International Agency for Research on Cancer, Lyon, France; National Cancer Institute, United States of America

## Abstract

**Background:**

Incidence-based mortality modelling comparing the risk of breast cancer death in screened and unscreened women in nine Swedish counties has suggested a 39% risk reduction in women 40 to 69 years old after introduction of mammography screening in the 1980s and 1990s.

**Objective:**

We evaluated changes in breast cancer mortality in the same nine Swedish counties using a model approach based on official Swedish breast cancer mortality statistics, robust to effects of over-diagnosis and treatment changes. Using mortality data from the NordCan database from 1974 until 2003, we estimated the change in breast cancer mortality before and after introduction of mammography screening in at least the 13 years that followed screening start.

**Results:**

Breast mortality decreased by 16% (95% CI: 9 to 22%) in women 40 to 69, and by 11% (95% CI: 2 to 20%) in women 40 to 79 years of age.

**Discussion:**

Without individual data it is impossible to completely separate the effects of improved treatment and health service organisation from that of screening, which would bias our results in favour of screening. There will also be some contamination of post-screening mortality from breast cancer diagnosed prior to screening, beyond our attempts to adjust for delayed benefit. This would bias against screening. However, our estimates from publicly available data suggest considerably lower benefits than estimates based on comparison of screened versus non-screened women.

## Introduction

Swedish randomized trials of mammography screening (MMS) have shown decreases in breast cancer mortality of 21% (RR = 0.79; 95% CI: 0.70–0.89) in women 40 years old and more, after a median follow-up time of 6.5 years (range 3.0 to 18.1) [1]. The greatest reduction of relative risk of breast cancer death, by 32% (RR = 0.68; 95% CI: 0.59–0.80), was reported in the Two-County Trial after 20 years of follow-up [2].

Attempts to correct these results for the possible effect of confounding by self-selection and temporal trends in breast cancer incidence [3] suggest a reduction of 39% (RR = 0.61; 95% CI: 0.55 to 0.68). However changes in incidence, i.e. increasing trend, may be due to over-diagnosis in screened women. This is controversial, with estimates of the rate over-diagnosis ranging ranging from negligible [4] to 56% [5].

Mammography screening was introduced throughout Sweden during the 1980s and 1990s. Whether results of Swedish randomized trials were reproduced when MMS was implemented in the general Swedish population has been addressed by several studies and estimated reduction of breast cancer mortality has bee estimated to be between 20% and 39% [6]–[11]. More recent studies compared incidence-based breast cancer mortality before and after the introduction of screening, i.e., breast cancer deaths in women diagnosed with breast cancer during a pre-defined period before and after introduction of screening were compared, irrespective of their participation in screening. Studies using incidence-based mortality were conducted in Darlana and Kopparberg counties, which were part of the Two-counties Study [12], and in nine other counties where screening was implemented [13].

As the attendance of Swedish women for MMS in the nine counties was high, from 70% to 88% [12], [14], a reduction of mortality of the magnitude predicted by the trials should be discernible in the breast cancer mortality data. The objective of this study was to assess changes in breast cancer mortality after the introduction of MMS in the same nine Swedish counties, using data available in the NordCan database [15]. We used the variation of starting dates for screening by county to separate screening effects from temporal trends in incidence.

While it would be desirable to extend the study to the twelve counties not included in the SOSSEG report [14], this was impossible because of the absence of available information on starting times and screening participation rates in any of those counties [16].

## Methods

Region-level aggregated data on breast cancer in Swedish women were obtained from the PC version of the NordCan database. NORDCAN is a data base providing 30 years of data (1974–2003) on mortality statistics from 41 major cancers in 81 regions in the Nordic countries [15]. Mortality data are obtained from national death registration systems.

From the most recent reports (13), we selected counties (with year of start of screening) Dalarna (1980), Gävleborg (1985), Norrbotten (1989), Örebro (1988), Stockholm (1990), Södermanland (1990), Uppsala (1990), Västernorrland (1990), and Västmanland (1990). The variation in start dates made it feasible to separate the effect of screening from nation-wide secular trends. Assuming that treatment improvements were not correlated with the date of introduction of screening, it should also be possible to separate screening and treatment effects.

Breast cancer mortality was modelled by Poisson regression, using the following model:
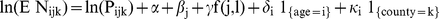



where


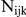
 number of cases in ith age group, year j, county k


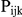
 number of person-years in ith age group, year j, county k


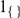
 indicator function (of age group or of county)


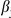
 start year of screening for region
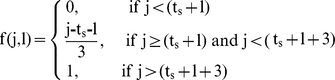



l lead time

α intercept term

β log relative risk associated with secular trend

γ log relative risk associated with screening

δ log relative risk associated with age group i

κ log relative risk associated with county k

The temporal trend coefficient β is intended to capture trends not correlated across counties with the dates of introduction of screening. We would expect that both changes in treatment and increases of incidence due to risk-factor changes should be captured by this term. In contrast, incidence changes due to screening related over-diagnosis are expected to be correlated with the screening start dates.

The role of the function *f(j,l)* is to take into account the lead time after the start of screening, and the impact of mortality from cases diagnosed prior to the introduction of screening. If *l* is the lead time, then no mortality benefit is seen before *l* years after screening, since we assume no breast cancer death can precede clinical detection. The lead time is important to be considered when assessing time from diagnosis of breast cancer to death from breast cancer, as lead time effect increases time to death when the cancer is screen detected as compared to when the cancer is symptomatic or clinically detected. We used a lead time of three years in our model [3], [17]. Lead time should not be confused with the separate issue of lead-time bias, which is of concern for studies of incidence or survival time.

It is also necessary to allow for the progressive uptake of screening, since not all women were screened immediately, and to account for variability in detection, timing of screening relative to cancer development, and rate of disease progression. Therefore we assumed that the screening benefit increased linearly from zero at *l* years after the start of screening, until the full benefit was seen at *l+3* years, i.e., the lead time plus 3 years. As a check that we were not creating the effect artificially in our model, we also applied it to mortality from non-breast cancer. All modelling was carried out using the R language (2.7.0) (http://www.R-project.org).

## Results

**Figure 1:**
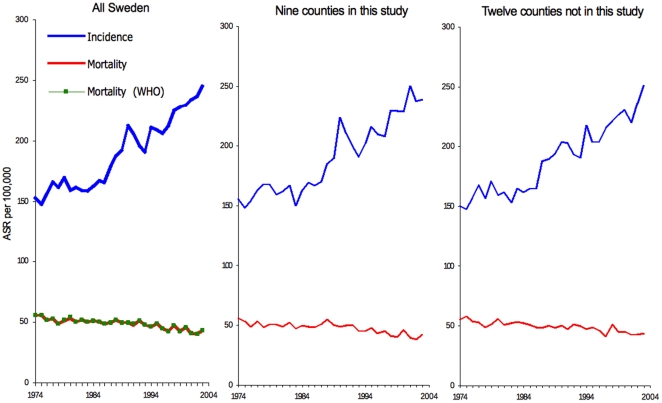
Breast cancer incidence and mortality in Swedish women 40 to 79 years old, 1974–2004.

Temporal trends for breast cancer incidence and mortality in Sweden are displayed in Figure 1. Population implementation of MMS started in 1980 in Dalarna county, and between 1985 and 1990 in the eight other counties [14]. The increase in breast cancer incidence is mainly noticeable after 1985, in counties included or not in the present study. In 2003, i.e., 13 years after last year of introduction of MMS, breast cancer incidence appeared to still be increasing (Figure 1). The secular monotone downward trend in breast cancer mortality since 1972 [18] was similar in counties included in the study and in those not included. Trends in breast cancer mortality using WHO data or NordCan data were sufficiently similar that they cannot be distinguished graphically, indicating similarity of data sent by the Swedish authorities to WHO and mortality data used by the NordCan project.

### Mortality Modelling

Using the 3 year lead time estimate, reduction of breast cancer mortality after the start of screening was 16% (RR = 0.84; 95% CI: 0.78–0.91 ) in women 40 to 69 years of age, and 11% (RR = 0.89; 95% CI: 0.80–0.98) in women 70 to 79 years of age. Inclusion or exclusion of county as a categorical explanatory variable in models did not materially change the results.

**Figure 2:**
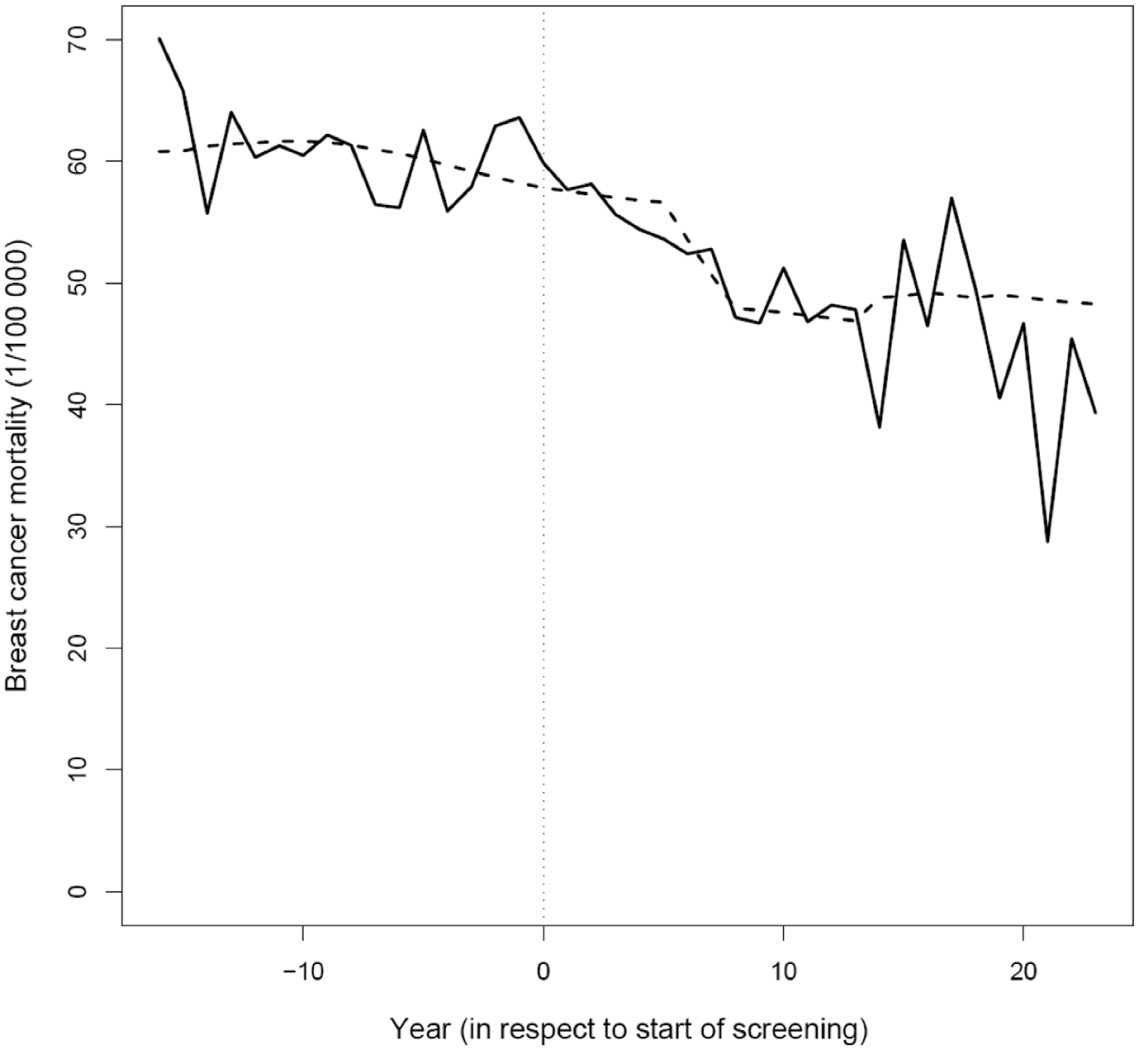
Mortality rate of breast cancer in Sweden in women 40–79 years of age in nine counties, according to time of screening start ( = 0) in each county. The continuous line is the crude mortality rate, and dashed line is mortality rate adjusted for age, secular trend, and screening effect.

Observed and adjusted breast cancer mortality data for all nine counties are summarized in Figure 2. A decrease in breast cancer mortality is present about 3 years after start of screening, followed by a stabilization of rates.

A similar modelling exercise using mortality for cancers other than breast cancer (Table 1) showed no significant effect of time after the start of MMS.

**Table 1:** Mortality of breast cancer (BC), and of other cancers in Swedish women from 1974 to 2003.

Age group	County	Person years(100 000)	Number of BC	BC deaths	Other cancer deaths
**(a) Age 40 to 69**	Stockholm	85.87	17978	4055	15891
	Dalarna	15.31	2931	648	2791
	Gävleborg	15.72	2593	684	3164
	Norrbotten	13.80	2086	498	2299
	Örebro	14.57	2532	675	2737
	Södermanland	13.58	2559	638	2637
	Uppsala	13.61	2428	571	2246
	Västernorrland	14.35	2596	669	2690
	Västmanland	13.07	2418	571	2543
**(b) Age 40 to 79**	Stockholm	105.91	23824	6190	30366
	Dalarna	19.57	3978	1032	5580
	Gävleborg	20.04	3617	1105	6342
	Norrbotten	16.89	2714	744	4195
	Örebro	18.55	3585	1074	5423
	Södermanland	17.03	3554	968	5047
	Uppsala	16.78	3257	867	4331
	Västernorrland	18.22	3677	1065	5496
	Västmanland	16.18	3272	881	4794

## Discussion

According to our descriptive epidemiology analyses, in women 40 to 69 years of age, the introduction of MMS in nine Swedish counties was associated with a 16% reduction of breast cancer mortality.

Using an incidence-based mortality method, the Swedish Organised Service Screening Evaluation Group (SOSSEG) conducted an evaluation based on comparison between women attending and not attending screening. Women not attending screening may have a less healthy lifestyle, to present with more advanced cancer and to have higher breast cancer mortality than women attending screening. In principle, failure to take into account the greater mortality of women not attending screening may lead to unbalanced comparison, and overestimation of the reduction of mortality to be expected from screening.

A companion paper to (13) using data from the same 9 counties using Poisson regression models assessed the risk of death from breast cancer in screened compared with unscreened women [3]. A reduction of 42% (95% CI: 38 to 47%) in breast cancer mortality was found for screened women, after adjusting for (i) changes in breast cancer fatality rate due to improving treatments, (ii) for lead time–corrected follow-up time, and (iii) and for the temporal trend of change in incidence among non screened women before and after start of screening. The reduction was 39% (RR = 0.61; 95% CI: 0.55–0.68) after further adjustment for self-selection.

The screening coverage in counties included in the SOSSEG study was 78% [3], and therefore the expected reduction in mortality for all women 50 to 69 years of age was approximately (39% * 0.78) = 30%. If we further correct for the self-selection effect (women attending screening contribute slightly less than their share of deaths), this decreases by a fraction 39/42 to 28%. Our estimate of 16% should be compared to this.

The reasons for the substantial discrepancy between our estimate and those of the Swedish group [3] follow from differences in model assumptions, model parameters, adjustment procedures and possibly data. The Swedish group modelled the risk of breast cancer death associated with being screened, whereas we modelled breast cancer mortality rates of the entire population eligible for screening.

One difference in approach is the correction for the change in incidence after and before the start of screening. This was done explicitly by the Swedish Group [3], which could lead to an over-estimate of the benefit of screening if there is screening-related over-diagnosis. There is relatively strong evidence that breast cancer incidence as much increased after screening introduction, which has lead to recognize overdiagnosis as being a major side effect of screening [19]–[23]. Our model seeks to distinguish temporal effects on mortality (β in our model) from screening by virtue of the varying dates of screening introduction by county. We felt this approach was preferable since incidence was already increasing faster than mortality [18] and it allows partial correction for both changes of incidence and treatment-related improvements in survival. Furthermore, in Sweden, breast mortality stabilized and started to decrease well before mammography screening programmes existed (see Figure 1).

Differences in data used by studies could be another possible reason for the difference in results. Breast cancer mortality data for Sweden in NordCan database are similar to those present in the WHO mortality database. We could compare numbers of breast cancer deaths in our and in the SOSSEG study (14) for the same period of time and age range for six counties (Table 2). On average, death counts used in the SOSSEG study [3] were 12% lower than that from the NordCan database. We are not aware of changes in death certification process in Sweden after 1980 likely to explain this discrepancy.

**Table 2:** Number of breast cancer deaths in women 40 to 69 years old in six Swedish counties according to two sources of data (3; 15).

		NordCan 2008		SOSSEG 2006b		
County	Period	Average population	Breast cancer deaths	Average population	Breast cancer deaths	% difference
Norrbotten	1976–2001	46 018	422	46 016	419	−1
Örebro	1979–2001	48 317	512	48 502	424	−17
Södermanland	1979–2000	45 245	466	45 445	384	−18
Uppsala	1985–2001	46 241	333	45 259	250	−25
Västernorrland	1974–2001	47 892	632	47 633	638	1
Västvmanland	1979–2001	46 110	436	46 115	352	−19
All 6 counties		279 824	2 801	278 970	2 467	−12

It remains also to determine if the reduction in breast cancer mortality we found after the start of screening was due to mammography screening itself, or to generalization of efficient cancer treatment that took place end of the 1980s, or to greater breast awareness and improved management of breast cancer patients induced by the screening programme (e.g., because of reorganization of clinical work or greater access to efficient treatments). The influence of treatment and of awareness in mortality reductions has been stressed by model approaches showing the key role of treatments [24]. In addition, in many high income countries, mortality reductions started well before or around the year screening started and these reductions were mainly observed in women less than 50 years of age even in countries where screening of young women is rare [25]. Our model attempts to correct for log-linear temporal trends in mortality, whether due to treatment or risk-factor induced changes in incidence. However it will not effectively capture step changes correlated to screening as would be expected from the above factors. This question has been addressed by the study by Kalager *et. al.* of mammographic screening in Norway (20), by comparing both screened and unscreened women to historic controls. They concluded that two-thirds of the reduction in mortality was likely due to non-screening factors, with screening itself generating a 10% (95% CI −4 to 24%) reduction in mortality. We would echo their conclusion: that the benefit of mammographic screening in isolation is modest, and that the larger estimates of benefit may be due to improved integration of treatment associated with screening.
